# Construct validity of the Chelsea critical care physical assessment tool: an observational study of recovery from critical illness

**DOI:** 10.1186/cc13801

**Published:** 2014-03-27

**Authors:** Evelyn J Corner, Neil Soni, Jonathan M Handy, Stephen J Brett

**Affiliations:** 1 Clinical Lead Respiratory Physiotherapist and Clinical Research Fellow, Chelsea and Westminster NHS Foundation Trust, Chelsea and Westminster Hospital, 369 Fulham Road, London SW10 9NH, England; 2 Imperial College London, London, UK; 3 Consultant in Intensive Care Medicine and Honorary Senior Lecturer, Chelsea and Westminster NHS Foundation Trust, Chelsea and Westminster Hospital, 369 Fulham Road, London SW10 9NH, England; 4 Consultant in Intensive Care Medicine and Reader in Critical Care, Centre for Perioperative Medicine and Critical Care Research, Imperial College NHS Healthcare Trust, Hammersmith Hospital, Du Cane Road, London W12 0HS, England, UK

## Abstract

**Introduction:**

Intensive care unit-acquired weakness (ICU-AW) is common in survivors of critical illness, resulting in global weakness and functional deficit. Although ICU-AW is well described subjectively in the literature, the value of objective measures has yet to be established. This project aimed to evaluate the construct validity of the Chelsea Critical Care Physical Assessment tool (CPAx) by analyzing the association between CPAx scores and hospital-discharge location, as a measure of functional outcome.

**Methods:**

The CPAx was integrated into practice as a service-improvement initiative in an 11-bed intensive care unit (ICU). For patients admitted for more than 48 hours, between 10 May 2010 and 13 November 2013, the last CPAx score within 24 hours of step down from the ICU or death was recorded (*n* = 499). At hospital discharge, patients were separated into seven categories, based on continued rehabilitation and care needs. Descriptive statistics were used to explore the association between ICU discharge CPAx score and hospital-discharge location.

**Results:**

Of the 499 patients, 171 (34.3%) returned home with no ongoing rehabilitation or care input; 131 (26.2%) required community support; 28 (5.6%) went to inpatient rehabilitation for <6 weeks; and 25 (5.0%) went to inpatient rehabilitation for >6 weeks; 27 (5.4%) required nursing home level of care; 80 (16.0%) died in the ICU, and 37 (7.4%) died in hospital. A significant difference was found in the median CPAx score between groups (*P* < 0.0001). Four patients (0.8%) scored full marks (50) on the CPAx, all of whom went home with no ongoing needs; 16 patients (3.2%) scored 0 on the CPAx, all of whom died within 24 hours. A 0.8% ceiling effect and a 3.2% floor effect of the CPAx is found in the ICU. Compliance with completion of the CPAx stabilized at 78% of all ICU admissions.

**Conclusion:**

The CPAx score at ICU discharge has displayed construct validity by crudely discriminating between groups with different functional needs at hospital discharge. The CPAx has a limited floor and ceiling effect in survivors of critical illness. A significant proportion of patients had a requirement for postdischarge care and rehabilitation.

## Introduction

For many, critical illness is catastrophic and, even for survivors, can be life changing. Multiorgan failure, along with its associated immobility and inflammatory state, leads to rapid and significant muscle loss, which can reach up to 15% within 7 days [1,2]. This is due to a reduction in protein synthesis and increased protein breakdown, as well as myofibril necrosis [1].

These pathophysiologic changes lead to measurable impairment of muscle strength associated with disability and functional decline, often coupled with a reduction in health-related quality of life [3]. This syndrome is commonly known as Intensive Care Unit-Acquired Weakness (ICU-AW). Research has shown that ICU-AW affects anywhere between 25% and 100% of critically ill patients [3]. With around 110,000 patients admitted to ICUs in England and Wales annually, this public health issue that extends well beyond the walls of the ICU [4,5], and we have no reason to conclude that this would be different in other countries.

The development of measurement tools has paved the way to facilitate both communication of patient description and assessment of interventions and outcome. In rehabilitation from critical illness, despite the obvious importance of identifying interventions so that recovery can be optimized, an incomplete development of tools has occurred to measure and describe levels of functional impairment [6,7]; still lacking is a simple means of measuring functional recovery from critical illness that can be used both to facilitate communication and as a potential outcome measure.

Of the measurement systems currently available for the assessment of ICU-AW, The Medical Research Council (MRC) sumscore has been commonly used [8,9]. This is an impairment-specific volitional test that involves the manual grading of strength in 12 muscle groups. However, the volitional nature of the MRC sumscore requires the patient to be cooperative, which is frequently problematic in the ICU cohort. Additionally, some components of the MRC score are subjective, and predictive validity for hospital length of stay and mortality is modest [10,11].

In the analysis of ICU-AW, it may be more appropriate to use a functional measure instead of an impairment-specific tool, as function is a manifestation of a number of different qualities, such as strength, cognition, alertness, balance, and so on. Hence using a functional measure would reduce the impact of volition on the test’s validity, and potentially may portray a more clinically relevant picture of the patients’ abilities. A number of different functional measures are currently under development.

The Physical Function ICU Test (PFIT) was developed in Australia, in a tertiary referral center for long-term tracheostomy patients [12]. This was later modified into the PFIT-score (PFIT-s) using RASCH analysis and tested in an acute ICU. Psychometrically, the PFIT-s has demonstrated validity in the critical care population; however, the large floor and ceiling effects on the ICU may limit utility [13].

The University of Rochester Acute Care Evaluation (URACE) [14] and the Functional Status Score for Intensive Care (FSS-ICU) [15] are both functional scoring systems from North America in the early stages of development. Both are potentially useful; however, they are very much in their infancy, and further psychometric evaluation is essential.

**Figure 1:**
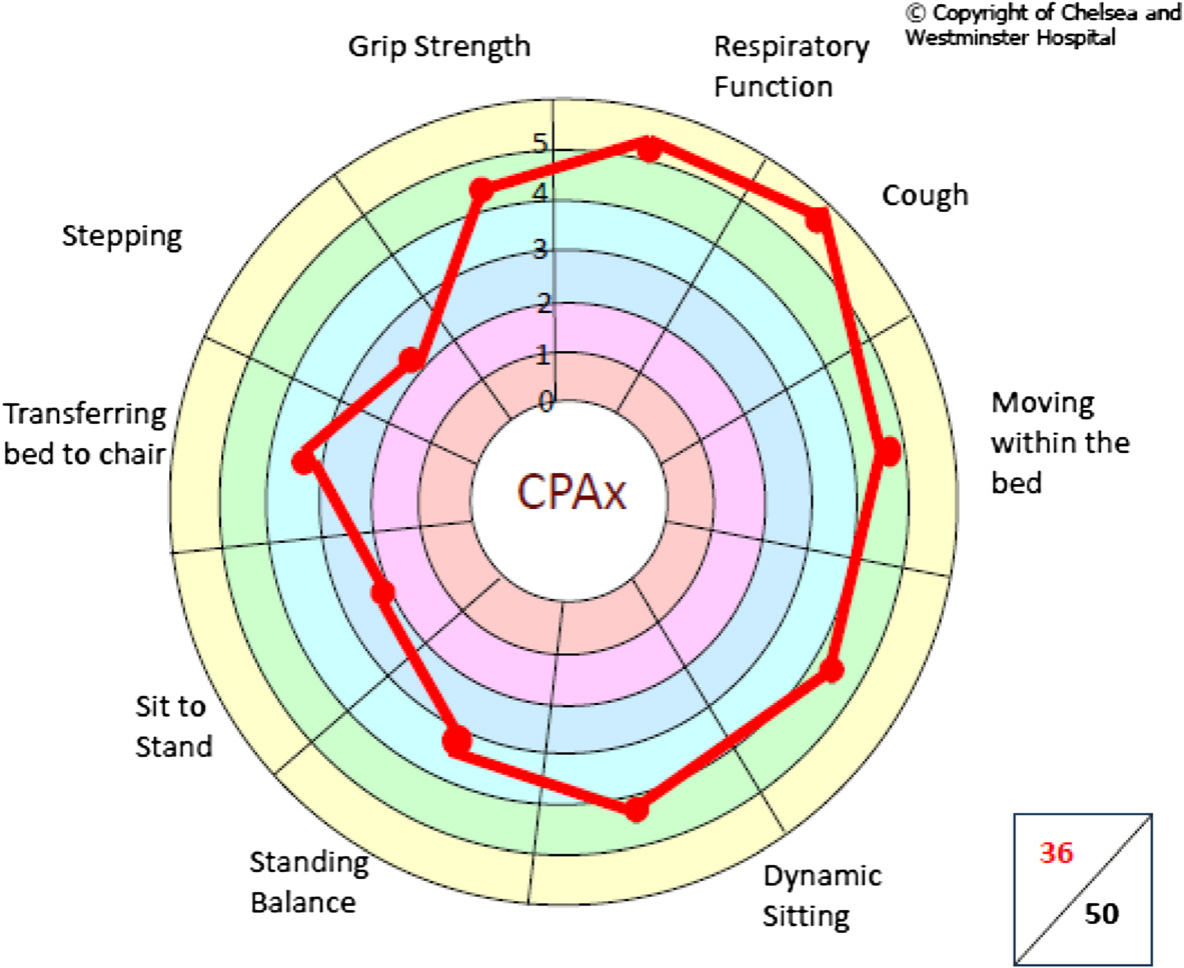
**Example of the CPAx radar chart.** This image demonstrates that this patient’s respiratory function, cough, and bed mobility are strong, and his rehabilitation should be tailored to work on gait reeducation, and on transferring from bed to chair, and from sit to stand.

In March 2009, development began of the Chelsea Critical Care Physical Assessment tool (CPAx), which is a numeric and pictorial composite of 10 commonly assessed components of physical function graded on a 6-point Guttman Scale, from complete dependence to independence (0 to 5). The component scores are added to give an overall score out of 50; 0 being the most-dependent patient, and 50 representing full independence. The score is also plotted on a “radar” chart, giving a rapid pictorial impression of patients’ functions and highlighting problem areas (Figure 1).

The CPAx was developed by using classic test theory through an iterative process including content-validity questionnaires, an expert focus group, service-user feedback, and an extensive pilot study on a cohort of 33 ICU patients [16]. This allowed proof of concept to be established through convergent validation and basic interrater reliability testing, which in turn provided a cogent argument for investigating the CPAx further. A copy of the CPAx tool can be found in Additional file 1.

The aim of this study was to explore further the construct validity of the CPAx by analyzing the association between CPAx scores on ICU discharge and hospital-discharge location. If the CPAx is to display construct validity, then higher CPAx scores at ICU discharge should be associated with better functional outcome in terms of rehabilitation/care needs at hospital discharge, crudely defined by discharge location.

Secondary aims were to analyze the usability of the CPAx at the test center, through compliance with completion of CPAx scores; to analyze floor and ceiling effects of the CPAx in the ICU; and to explore future level-of-care needs in critical care survivors.

## Methods

Data were collected from an 11-bedded general (mixed medical-surgical, levels 2 and 3) ICU in central London over a 42-month time period from 10 May 2010 to 13 November 2013. The physiotherapy service adopts a model of daily review during which the physiotherapists’ role includes both respiratory and physical therapy (that is, exercise and functional rehabilitation).

The CPAx was developed at this center and embedded into clinical practice as part of quality-improvement initiative to improve compliance with the comprehensive clinical-assessment component of the NICE guidelines for Rehabilitation after Critical Illness [17]. Hence this project was categorized as service evaluation by the local Research and Development Department and the National Research Ethics Service, and was considered exempt from the need for specific research ethics review. No patient consent was required for this study, as all data were anonymous and routinely collected.

All physiotherapists were taught how to use the CPAx tool by the primary developer (EC) in a case-based tutorial. The CPAx has previously been shown to have good interrater reliability, so no further testing was completed as part of this study [16]. After training, the CPAx was integrated into clinical practice, through an iterative process of plan do study act (PDSA) cycles, adopted from service-improvement methods. Compliance with completion of the score was monitored regularly as the percentage of all patients admitted with a completed CPAx score. When compliance was low and/or dropped, the data-recording strategies were modified until use of the CPAx score was embedded into clinical practice.

The clinical aim was for all patients staying in the ICU for more than 48 hours to be scored throughout their stay by using the CPAx at least 3 times per week, unless there was a reason for exemption (for example, they did not require physiotherapy input). The ICU physiotherapists collated the patients’ last CPAx scores taken within 24 hours of step down from ICU or death. The ward-based physiotherapist or occupational therapist then recorded the patients’ discharge location as part of their discharge-planning process. The discharge locations were separated into five survival categories in a hierarchic manner, and two nonsurvival categories, listed in Table 1.

**Table 1:** Hospital-discharge categories

Survival categories	1. Home with no rehabilitation needs, considered the optimal outcome	No community services accessed
	2. Home with community support, this may vary from a full package of care to outpatient physiotherapy	Package of care
		Integrated care team
		Domiciliary therapy
		Outpatient therapy
	3. A short-stay inpatient-rehabilitation facility (<6 weeks)	An inpatient facility that had a maximum length of stay of 6 weeks.
	4. A long-stay rehabilitation facility (>6 weeks)	An inpatient facility that had an expected length of stay of more than 6 weeks.
	5. Nursing-home level of care	Ongoing daily nursing needs in a nursing home environment or home with a maximal-care package, including a hospital bed and hoist transfers.
Nonsurvival categories	1. Nonsurvival from ICU	
	2. Nonsurvival from hospital	

The ward and ICU physiotherapist were asked not to discuss the CPAx scores of the patient on ICU discharge; however, no formal measures were taken to guarantee blinding of responsible medical or surgical multidisciplinary teams. It is unlikely, however, that the CPAx score would have influenced the patients’ final discharge locations, as this would have been based on clinical need in absence of prognostic evidence of the CPAx tool at the time of the study.

Patients were excluded if they fitted the following criteria: ICU stay < less than 48 hours; no recorded CPAx score within 24 hours of discharge from ICU; discharge location unclear.

### Statistical analysis

Statistical analysis was performed with Excel (version 2010; Microsoft Corporation, Seattle, WA, USA) and Prism (version 5; GraphPad Software, San Diego, CA, USA). Data were assessed for normality by using the D’Agostino and Pearson omnibus normality test and are presented as mean (±standard deviation) or median (interquartile range); parametric or non-parametric equivalent tests were used as appropriate. As the CPAx is an ordinal scale, medians (interquartile ranges) of the CPAx score are reported. Statistical differences between discharge locations were assessed by analysis of variance (Kruskal-Wallace), Dunn multiple comparison test was used to analyze the individual differences between groups *post hoc*. The primary level of significance was set at *P* < 0.05 adjusted for multiple comparisons.

## Results

From 10 May 2010 to 13 November 2013, 1,524 patients were admitted to the ICU. Demographic data of all 1,524 patients are reported in Table 2.

**Table 2:** **Demographic data for all patients admitted during the study period (*n*** **= 1,524): data are presented as number (percentage) and mean (SD) or median (IQR)**

**Age in years**	**58 (19.1)**
Male	773 (50.6%)
Female	751 (49.2%)
Number receiving mechanical ventilation	691 (45.3%)
Mean ICU length of stay in days	6.8 (12.9)
Median ICU length of stay in days	2.6 (1–7.1)
Mean hospital length of stay in days	31.8 (77.7)
Median hospital length of stay in days	14 (6–30)
Survival at ICU discharge	1,331 (87.2%)
Mortality in ICU	188 (12.3%)
Median admission APACHE II	15 (10–20)
*Referring specialty*	
Surgery	673 (44.1%)
Obstetrics and gynecology	88 (5.8%)
Medicine	485 (31.8%)
HIV/GUM	48 (3.1%)
Interhospital critical care transfer	94 (6.2%)
Other	139 (9.1%)

**Figure 2:**
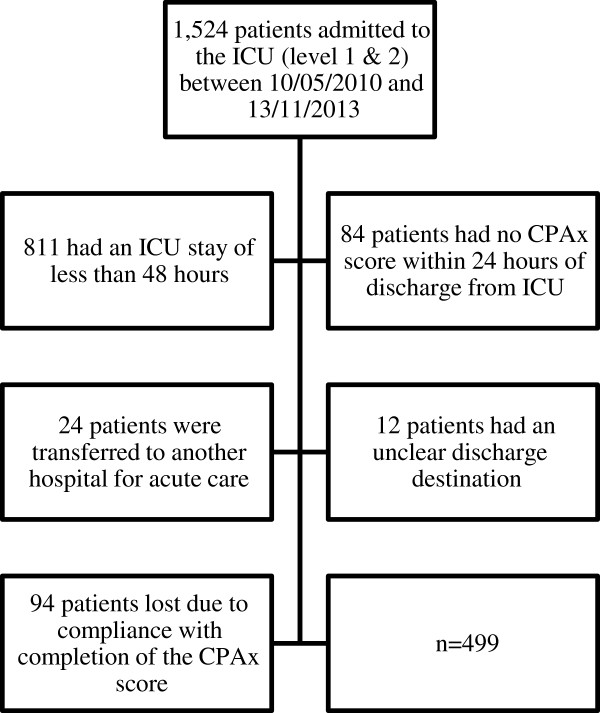
Consort diagram.

Of the 1,524 patients admitted during this period, 836 patients met the exclusion criteria. Final data are reported for 499 patients. Demographic data for the 499 patients included in the analysis are reported in Table 3, with subgroup demographics for patients separated by discharge location. Attrition information is displayed as a consort diagram in Figure 2.

**Table 3:** Demographic-subgroup analysis for each discharge category and the study population

	**Whole population**	**Home with no rehab needs**	**Home with community support**	**Short-stay rehabilitation facility (<6 weeks)**	**Long-stay rehabilitation facility (>6 weeks)**	**Nursing home level of care**	**Nonsurvival from ICU**	**Nonsurvival from hospital**
*N* [%]	499	171 [34.3%]	131 [26.3%]	28 [5.6%]	25 [5.0%]	27 [5.4%]	80 [16.0%]	37 [7.4%]
Age (mean (SD))	62.3 [18.31]	56.2 [17.41]	63.4 [18.33]	72.5 [12.1]	56.0 [15.8]	56.6 [24.56]	68.4 [16.37]	72 [14.04]
APACHE II (median (IQR))	16 [10–20]	14 [11-18]	16 [13–21]	17 [13–20]	17 [11–20]	17 [16–24]	22 [18–24]	18 [16–22]
Mean ICU length of stay (SD)	11.54 [16.18]	6.44 [5.48]	11.27 [12.86]	21.7 [26.6]	33.28 [41.6]	15.0 [17.5]	13.0 [12.3]	8.74 [7.8]
Median ICU length of stay (days (IQR))	6 [4-12]	4.5 [3-8]	6.8 [3-14]	9 [4–31]	14.2 [9–43]	7.1 [5–19]	9.3 [4-15]	5.9 [4-11]
Diagnosis (*n* (1%))								
- Cardiac arrest	19 [3.8%]	1 [0.6%]	5 [3.8%]	0	0	2 [7.4%]	10 [12.5%]	1 [2.7%]
- Chronic cardiovascular disease	21 [4.2%]	2 [1.2%]	11 [8.4%]	3 [10.7%]	1 [4%]	2 [7.4%]	0	2 [7.4%]
- Cardiovascular dysfunction	19 [3.8%]	7 [4.1%]	4 [3.1%]	3 [10.7%]	0	1 [3.7%]	4 [5%]	0
- Cardiovascular monitoring	9 [1.8%]	4 [2.3%]	2 [1.5%]	0	0	0	1 [1,3%]	2 [7.4%]
- Diabetic ketoacidosis	3 [0.6%]	3 [1.8%]	0 [0%]	0	0	0	0	0
- Gastrointestinal dysfunction	67 [13.4%]	25 [14.6%]	20 [12.3%]	4 [14.3%]	1 [4%]	3 [11.1%]	8 [10%]	6 [22.2%]
- Gastrointestinal surgery	38 [7.6%]	18 [10.5%]	12 [9.2%]	2 [7.1%]	0	2 [7.4%]	4 [5%]	0
- Metabolic/Renal system dysfunction	39 [7.8%]	11 [6.4%]	11 [8.4%]	6 [21.4%]	3 [12%]	0	6 [7.5%]	2 [7.4%]
- Neurological system dysfunction	30 [6.0%]	5 [2.3%]	8 [6.1%]	1 [3.6%]	4 [16%]	3 [11.1%]	6 [7.5%]	3 [11.1%]
- Poisoning	4 [0.8%]	4 [2.3%]	0	0	0	0	0	0
- Hemorrhage	11 [2.2%]	7 [4.1%]	0	0	0	0	1[1.3%]	3 [11.1%]
- Respiratory system dysfunction	148 [29.7%]	54 [32%]	37 [28.3%]	6 [21.4%]	5 [20%]	9 [33.3%]	24 [30%]	13 [48.1%]
- Sepsis	70 [14.0%]	25 [14.6%]	16 [12.2%]	2 [7.1%]	7 [28%]	1 [3.7%]	14 [17.5%]	5 [18.5%]
- Trauma	10 [2.0%]	0	3 [2.3%]	1 [3.6%]	2 [8%]	4 [14.8%]	0 [0%]	0
- Other	11 [2.2%]	5 [2.9%]	2 [1.5%]	0	2 [8%]	0	2 [2.5%]	0

These data were normally distributed in the short-stay and long-stay rehabilitation groups; in the remaining five groups, data were skewed, so all data were treated as nonparametric.

### Construct validity

**Figure 3:**
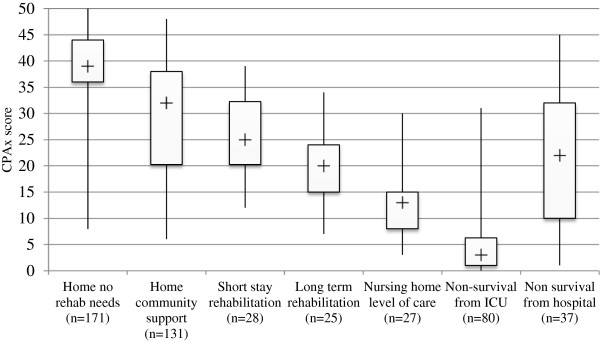
**Box-and-whisker diagram demonstrating the median CPAx score for patients divided by discharge location (*n*** **= 498).**

The median and interquartile range of CPAx scores for patients when grouped by discharge location is summarized in Figure 3.

Analysis of variance showed statistically significant differences in the median CPAx scores between the seven discharge groups (H (2) = 311.4, *P* < 0.0001). *Post hoc* analysis of the difference in median CPAx score between each individual discharge group is reported in Table 4.

**Table 4:** ***Post hoc *** **analysis of between-group differences**

	**Home with no rehabilitation needs**	**Home with community support**	**Short-stay rehabilitation (<6 weeks)**	**Long-stay rehabilitation (>6 weeks)**	**Nursing-home level of care**	**Nonsurvival from ICU**
Home with community support	*P* < 0.05					
Short-stay rehabilitation (<6 weeks)	*P* < 0.05	ns				
Long-stay rehabilitation (>6 weeks)	*P* < 0.05	*P* < 0.05	ns			
Nursing-home level of care	*P* < 0.05	*P* < 0.05	ns	ns		
Nonsurvival from ICU	*P* < 0.05	*P* < 0.05	P < 0.05	*P* < 0.05	ns	
Nonsurvival from hospital	*P* < 0.05	ns	ns	ns	ns	*P* < 0.05

### Usability

During the study period, the CPAx score was calculated a total of 6,309 times with an average of 7.5 CPAx scores per patient. Our target was a minimum of three CPAx assessments per patient, per week. The average ICU length of stay for our cohort was 11.54 days, so ideally, patients would have had a CPAx score equated at least 5 times; hence the target number of CPAx assessments was exceeded.

**Figure 4:**
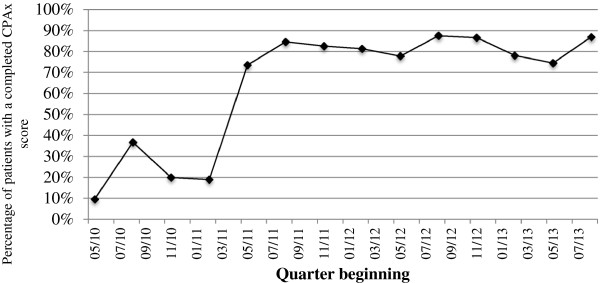
The percentage of all patients admitted to ICU with a completed CPAx score on a quarterly basis between May 2010 and November 2013.

In the initial stages of introducing the CPAx tool into clinical practice, compliance with completion of the CPAx was quite low; quarterly percentage compliance data are presented in Figure 4. Compliance is defined as the percentage of all ICU admissions with a completed CPAx score on a quarterly basis.

A number of PDSA cycles were completed to look at implementation strategies to improve compliance rates. This included training physiotherapists to use the CPAx effectively, and altering the method of data recording. Data-recording strategies began with a paper-based system, which yielded compliance rates averaging at around 10% from May 2010 to August 2010. The CPAx was then integrated onto the ICU observation charts, increasing average compliance to around 25% from September 2010 to April 2011. Finally, the CPAx was added to the local computerized notes system with a forcing function to ensure completion of the CPAx unless it was not clinically indicated; in this case, a password was entered after discussion with a senior physiotherapist. This improved compliance rates dramatically, with a consistent compliance since June 2011 until now, averaging 78% of all ICU admissions.

### Floor and ceiling effect

Four patients of 499 (0.8%) scored full marks of 50/50 on the CPAx score. These were all in the “home with no rehabilitation needs” group. Sixteen patients (3.2%) scored the lowest possible score of 0/50 on the CPAx score. All of these patients died within 24 hours of this score being recorded.

### Future care needs

Of the 499 patients, 171 (34.3%) returned home with no ongoing rehabilitation/care input. The remaining 210 (42.3%) survivors all required some form of ongoing rehabilitation or care: 131 (26.2%) required community support; 28 (5.6%) went to inpatient rehabilitation for 6 weeks or less; and 25 (5.0%) went to inpatient rehabilitation for more than 6 weeks; 27 (5.4%) required nursing-home level of care. For the nonsurvival groups, 80 (16.0%) died in the ICU, and 37 (7.4%) died before hospital discharge.

## Discussion

Construct validity is a method of validation frequently used in the absence of a gold-standard comparator and/or when measuring a construct that is difficult to observe [6]. It involves the development of hypotheses that a new scoring system or questionnaire will behave as expected in a specified population. For example, one would expect patients in a persistent vegetative state to have a lower Glasgow Coma Scale (GCS) score than patients in a general medical ward.

Although the difference in average CPAx score between groups did not reach statistical significance for all discharge categories, these data show clear associations between CPAx score at ICU discharge and final hospital-discharge locations. In the survival groups, those that went home with no rehabilitation input, which would be considered the optimal outcome, scored the highest on the CPAx score; and those requiring nursing-home level of care, arguably the poorest survival outcome, scored the lowest. This supports the hypothesis that the CPAx score at final assessment within critical care is associated with long-term recovery and rehabilitation trajectory, and thus has construct validity. Taken together with the previous validation work [16], it would seem reasonable to assume that the CPAx may have a role in the assessment, description, and monitoring of functional recovery in patients with ICU-AW.

On *post hoc* analysis, the individual differences between groups reached significance in only 12 of the 21 comparisons, with those returning home with no rehabilitation needs scoring significantly more than all other groups. The numbers available for detailed intergroup comparisons were insufficient to achieve statistical significance. However, we would contend that the trends demonstrated in Figure 3 support the construct validity of the CPAx tool as a measure associated with future recovery trajectory. The individual variation of recovery trajectory is substantial, so it would not be appropriate to use the ICU discharge CPAx score as an individual predictor of specific future destination, but may be a crude indicator of whether a patient is likely to need ongoing rehabilitation.

The variability in the CPAx scores for each discharge-location group might be explained by a number of factors: the heterogeneity of critical care patients; the level of perceived “rehabilitation potential”; the baseline level of function; and crucially, the level of family support and home setup available to the patient. For example, the patients that were discharged home with community support had the widest range of CPAx scores. Clinically this seems logical, as a large variation in the level of support can be provided in a home environment. This may vary from a large package of care for an elderly and frail patient, to an outpatient physiotherapy service for the more mobile.

Those discharged to a rehabilitation facility will be admitted only if they are perceived to have rehabilitation potential (that is, the ability to progress from their current functional level. Those considered to have reached their full potential are more likely to be discharged to a facility with appropriate support (for example, a nursing home or home in a modified environment with a package of care). This may explain the narrower range of CPAx scores for these groups.

Of note, the final location for discharge populations is likely to be directly related to the facilities available in local communities; hence, generalizing these data to other geographic locations should be done with extreme caution.

For the nonsurvival group, those that died in the ICU had the lowest CPAx score at their last assessment. Those that later went on to die in the ward scored surprisingly high on the CPAx score at ICU discharge. Subsequent analysis revealed that the average age and APACHE II scores for this group were at 72 and 18, respectively, and many of these patients died of unforeseen medical emergencies or were given palliation.

On subgroup analysis, there also appears to be an association between age, ICU length of stay, and APACHE II scores, with discharge location. As might be expected, those that return home with no ongoing rehabilitation were younger (mean, 56.2 years), had a shorter ICU length of stay (mean days (SD), 16.18) and lower APACHE II scores (median (IQR): 14 (11–18)). Those that went home with community support tended to be a little older than the first group (mean age, 63.4 years), sicker (APACHE II median (IQR): 16 (13–21), and had a longer mean length of stay (days (SD): 11.27 (12.86)); and this trend continues until the long-stay rehabilitation and nursing-home level-of-care categories, who included two of the youngest groups, averaging 56 years (SD, 15.8) and 56.6 years (SD, 24.56), respectively. Of note, these categories also had the highest proportion of neurology patients, which may help to explain the younger age bracket.

Furthermore, long-term rehabilitation facilities are sparse, so their admission criteria tend to be more specific, often favoring those considered to have more rehabilitation potential, which may be associated with a younger cohort. Those discharged to a nursing-home facility had one of the highest average APACHE II scores (median (IQR): 17 (16 to 24]); it is possible that older patients with significant functional limitation requiring long-term nursing care are less likely to survive until hospital discharge, so there may be an element of natural selection for the younger patient within this category. Furthermore, these findings may be associated with admission criteria to the ICU and previous functional level.

A number of other authors have published work on new scoring systems to measure function in critical care, of which the Physical Functional Test for use in the ICU-s (PFIT-s) has been through the most stringent psychometric analysis, demonstrating convergent, discriminant, and predictive validity. This testing also included Rasch analysis, allowing conversion of the score from an ordinal to an interval scale [13].

The PFIT-s combines assessment of shoulder and knee strength graded on the Oxford scale with cadence (steps/minute) and level of assistance. The content of this score is very different from the functional components in the CPAx score, focusing more on strength, endurance, and exercise capacity, than on function. This, and the volitional nature of the PFIT-s, may explain the 21.5% floor and 22.2% ceiling effect on critical care: an important limitation in its clinical and research application [13]. The floor and ceiling effect of the CPAx tool in the ICU is minimal, with only four of 499 patients scoring full marks, and 16 patients scoring 0; all of whom unfortunately died in the next 24 hours, suggesting that the CPAx is likely to be able to detect change throughout the spectrum of functional level seen in the ICU and thus may be a more useful measure at this time point. However, no scoring system will ever span the spectrum from complete dependence to full function; it may be more appropriate for a battery of scores to be used at different time points throughout the patient journey.

The Functional Status Score for ICU (FFS-ICU) is also in development [15]. The FSS-ICU has more similarities to the CPAx scale, in that it grades patients on a Guttman scale from 1 to 7, dependent on their level of assistance required, and it assesses five of the same functional tasks: ambulation, rolling, sitting, supine to sitting, and sit-to-stand transfers.

The FSS-ICU has shown similar associations with hospital discharge location as the CPAx in a smaller cohort of 101 patients in North America, which is unsurprising, given the similarities in the contents of the two tools. Notably, the investigators also showed a wide range of FSS-ICU scores for the five discharge categories that they used: home; inpatient-rehabilitation facility; skilled-nursing facility; hospice/long-term care; and transfer to short-stay hospital [15]. Although key differences in practice exist between North America and Europe, making difficult a direct comparison between results, this wide range of CPAx and FSS-ICU scores for each discharge group supports the suggestion that accurate prediction of hospital-discharge location is likely to be difficult. This is probably due to its interdependency on so many other factors (for example, social setup and support and premorbid functional level).

The concurrent but independent development of the CPAx and the FFS-ICU in different continents is supportive of the face and content validity of the basic common concepts behind both measures. Importantly, the differences between the two tools, the respiratory and cough components of the CPAx, probably reflect the difference in physiotherapy *versus* physical therapy practice between Europe and Australasia, and North America. In Europe and Australasia, the role of the respiratory therapist *and* physical therapist *practiced in North America is merged into one. As* physiotherapists have a key role in weaning from mechanical ventilation and airway clearance, the inclusion of the cough and respiratory-function components in the CPAx, and the exclusion of these components in the FFS-ICU, may indicate that each score is more suited to the professional environment in which they were developed. As respiratory muscle strength is closely associated with peripheral muscle strength and thus function, it is important that this be considered in a measurement scale designed for weaning patients.

These service-evaluation data have demonstrated that the CPAx can be implemented in a sustainable manner, with consistently around 78% of all ICU admissions receiving a comprehensive clinical assessment on the CPAx tool once established. Any patients not scored by using the CPAx will have been assessed for suitability by a senior clinician and actively excluded. The most common reason for this was that the patient was not requiring, or thus receiving any physiotherapy intervention; or because a full assessment could not be completed (for example, because they were receiving a medical intervention or declined assessment). The time taken to complete the CPAx score has not been formally evaluated; however, it is estimated from staff feedback to take an average of around 2 minutes; this is primarily because it was designed to allow a number to be attributed to what would be considered a standard physiotherapy assessment in the UK, suggesting that it is a simple and usable measure. The grip-strength component of the CPAx assessment is the only additional item to standard ICU physiotherapy assessments.

Importantly, these data also illustrate the impact that critical illness has had on our local community health services over the past 3-year period. An alarming 210 patients of the 499 in this population who were discharged alive required ongoing support and rehabilitation, demonstrating how ICU-AW is a public health issue that extends well beyond the walls of the ICU [5].

### Limitations

This was a service evaluation completed at one center in a predominantly affluent area of London. This may have had an impact on the case mix, although the demographic and diagnostic data look reasonably typical for a mixed UK critical care population [18]. There was no blinding of the physiotherapists making the clinical decisions about discharge location to the patients’ CPAx scores. It is unlikely that this would have influenced the discharge location, as discharge destination would be based on clinical need, and reliance on the CPAx score as a guide may have been unsafe.

Some missing data, and variable compliance with completion of the scores, were found predominantly from the early phases; the impact of this on the final results is likely to be small in a cohort of 499. Although only CPAx scores within 24 hours of ICU discharge were included, some CPAx scores were taken while the patient was still intubated; hence the validity of those specific scores as a reflection of the patients’ true function at ICU discharge may be questioned; if these scores had been excluded, it is likely to have narrowed the spread of data. Discharge from ICU is a loose outcome time point, as it is dependent on a number of factors (for example, bed availability and ward staffing).

The *post hoc* analysis of differences in average CPAx scores between groups did not reach statistical significance for all categories, which may limit the validity of the results; however, this is tempered by the clear association between CPAx scores and discharge location, the likeness of these data to clinical practice, and the crudity of discharge location as a measure of outcome.

With regard to implementation, it should be noted that this report describes the experience of implementation in the hospital in which the tool was developed and thus had strong local champions. However, the CPAx has now been sent out for adoption at 86 other institutions, many of which have, in their view, successfully embedded its use within practice.

## Conclusions

The data presented would support the contention that the CPAx has construct validity, and taken with previously published validation work, it would now seem reasonable to use this in practice, with the caveat that its performance requires continuing evaluation.

The CPAx score has shown strong associations with hospital-discharge location, as a surrogate for functional outcome; can be embedded into clinical practice sustainably; and it has a limited floor and ceiling effect on the ICU. Integration of the CPAx into clinical practice may help professionals to monitor and describe functional recovery from critical illness in a more objective manner, thus opening up communication between professionals and service users about patients’ functional recovery, or lack thereof.

Further studies are essential to investigate whether the CPAx will help to identify areas of rehabilitation need, thus assisting in physiotherapy assessment, treatment planning, and goal setting; further analysis of training and implementation; and whether the CPAx has sufficient psychometric properties to be used as a standardized outcome assessment in clinical practice and interventional research.

This work also demonstrates that the level of ongoing rehabilitation need in critical care survivors is substantial, illustrating just how catastrophic critical illness can be.

## Key messages

•The CPAx score can be embedded into clinical practice sustainably.

•The CPAx score shows strong associations with hospital-discharge location as surrogate for functional outcome, implying construct validity.

•The CPAx has a limited floor-and-ceiling effect on the ICU, also implying validity.

•The level of ongoing rehabilitation need in critical care patients’ survivors is substantial, illustrating just how catastrophic critical illness can be.

## Abbreviations

ANOVA: analysis of variance; CPAx: Chelsea Critical Care Physical Assessment tool; FSS-ICU: Functional Status Scale for ICU; ICU: Intensive care unit; ICU-AW: critical care unit acquired weakness; PDSA: Plan do study act; PFIT: Physical Functional Test for the ICU; PFIT-s: Interval Scored Physical Functional Test for the Intensive Care Unit; URACE: University of Rochester Acute Care Evaluation.

## Competing interests

The authors declare that they have no competing interests.

## Authors’ contributions

EC was the primary developer of the CPAx. All authors contributed to the conception and design of this study, data analysis and interpretation, draft and revised manuscript development, and approval of the final version for publication.

## Supplementary Material

Additional file 1 The CPAx tool and grip-strength reference table. Click here for file
