# Supplementary material for: Construct validity of the Chelsea critical care physical assessment tool: an observational study of recovery from critical illness
Source: Crit Care. 2014 Mar 27;18(2):R55. doi: 10.1186/cc13801 (PMC4056321; doi:10.1186/cc13801)
Supplement: Additional file 1 — The CPAx tool and grip-strength reference table. [file cc13801-S1.pdf]

The Chelsea Critical Care Physical Assessment tool (CPAx)

Aspect of Physicality	Level 0	Level 1	Level 2	Level 3	Level 4	Level 5
**Respiratory Function**	Complete ventilator dependence. Mandatory breaths only. May be fully sedated/ paralysed.	Ventilator dependence. Mandatory breaths with some spontaneous effort.	Spontaneously breathing with continuous invasive or non-invasive ventilatory support.	Spontaneously breathing with intermittent invasive or non-invasive ventilatory support **Or** continuous high flow oxygen (>15litres).	Receiving standard oxygen therapy (<15 litres).	Self-ventilating with no oxygen therapy.
**Cough**	Absent cough, may be fully sedated or paralysed.	Cough stimulated on deep suctioning only.	Weak ineffective voluntary cough, unable to clear independently e.g. requires deep suction.	Weak, partially effective voluntary cough, sometimes able to clear secretions e.g. requires yanker suctioning.	Effective cough, clearing secretions with airways clearance techniques.	Consistent effective voluntary cough, clearing secretions independently.
**Moving Within the Bed e.g. rolling.**	Unable, maybe fully sedated/ paralysed.	Initiates movement. Requires assistance ≥ 2 people (maximal).	Initiates movement. Requires assistance≥1 person (moderate).	Initiates movement. Requires assistance 1 person (minimal).	Independent in ≥3 seconds.	Independent in <3 seconds.
**Supine to Sitting on the Edge of the Bed.**	Unable/ Unstable.	Initiates movement. Requires assistance ≥ 2 people (maximal).	Initiates movement. Requires assistance≥1 person (moderate).	Initiates movement. Requires assistance 1 person (minimal).	Independent in ≥3 seconds.	Independent in <3 seconds.
**Dynamic Sitting (i.e. when sitting on the edge of the bed/unsupported sitting)**	Unable/ Unstable	Requires assistance ≥2 people (maximal).	Requires assistance≥1 person (moderate).	Requires assistance 1 person (minimal).	Independent with some dynamic sitting balance, i.e. able to alter trunk position within base of support.	Independent with full dynamic sitting balance, i.e. able to reach out of base of support.
**Standing Balance**	Unable/ unstable/ bedbound.	Tilt table or similar	Standing hoist or similar.	Dependant on frame, crutches or similar.	Independent without aides.	Independent without aids and full dynamic standing balance, i.e. able to reach out of base of support.
**Sit to Stand** (Starting position: ≤ 90 degrees hip flexion)	Unable/ Unstable.	Sit to stand with maximal assistance e.g. standing hoist or similar.	Sit to stand with moderate assistance e.g. 1-2 people.	Sit to stand with minimal assistance e.g. 1 person.	Sit to stand independently pushing through arms of the chair.	Sit to stand independently without upper limb involvement.
**Transferring from Bed to Chair.**	Unable/ Unstable.	Full hoist.	Standing hoist or similar.	Pivot transfer (no stepping) with mobility aid or physical	Stand and step transfer with mobility aid OR physical	Independent transfer without equipment.

© Copyright of Chelsea and Westminster NHS Foundation Trust (01/03/2010)

The Chelsea Critical Care Physical Assessment tool (CPAx)

				assistance.	assistance.	
**Stepping**	Unable/ Unstable.	Using a standing hoist, or similar.	Using mobility aids AND assistance > 1 person (moderate).	Using mobility aid AND assistance 1 person (minimal).	Using mobility aid OR assistance 1 (minimal).	Independent without aid.
**Grip Strength** (predicted mean for age and gender on the strongest hand.)	Unable to assess.	< 20%	< 40%	< 60%	< 80%	≥80%

© Copyright of Chelsea and Westminster NHS Foundation Trust (01/03/2010)

The Chelsea Critical Care Physical Assessment tool (CPAx)

Age	Men							Women						
	Hand	Mean	<20%	<40%	<60%	<80%	≥80%	Hand	Mean	<20%	<40%	<60%	<80%	≥80%
**15-19**	R	46.91	9.38	18.76	28.15	37.53	37.53	R	28.82	5.76	11.53	17.29	23.06	23.06
	L	42.13	8.43	16.85	25.28	33.70	33.70	L	24.98	5.00	9.99	14.99	19.98	19.98
**20-24**	R	48.15	9.63	19.26	28.89	38.52	38.52	R	28.33	5.67	11.33	17.00	22.66	22.66
	L	43.08	8.62	17.23	25.85	34.46	34.46	L	25.78	5.16	10.31	15.47	20.62	20.62
**25-29**	R	53.76	10.75	21.50	32.26	43.01	43.01	R	33.82	6.76	13.53	20.29	27.06	27.06
	L	48.60	9.72	19.44	29.16	38.88	38.88	L	30.31	6.06	12.12	18.19	24.25	24.25
**30-34**	R	52.63	10.53	21.05	31.58	42.10	42.10	R	33.97	6.79	13.59	20.38	27.18	27.18
	L	48.98	9.80	19.59	29.39	39.18	39.18	L	31.64	6.33	12.66	18.98	25.31	25.31
**35-39**	R	53.16	10.63	21.26	31.90	42.53	42.53	R	32.46	6.49	12.98	19.48	25.97	25.97
	L	51.75	10.35	20.70	31.05	41.40	41.40	L	29.77	5.95	11.91	17.86	23.82	23.82
**40-44**	R	55.49	11.10	22.20	33.29	44.39	44.39	R	30.34	6.07	12.14	18.20	24.27	24.27
	L	50.40	10.08	20.16	30.24	40.32	40.32	L	26.23	5.25	10.49	15.74	20.98	20.98
**45-49**	R	49.93	9.99	19.97	29.96	39.94	39.94	R	35.30	7.06	14.12	21.18	28.24	28.24
	L	48.94	9.79	19.58	29.36	39.15	39.15	L	32.06	6.41	12.82	19.24	25.65	25.65
**50-54**	R	48.40	9.68	19.36	29.04	38.72	38.72	R	28.37	5.67	11.35	17.02	22.70	22.70
	L	41.46	8.29	16.58	24.88	33.17	33.17	L	26.28	5.26	10.51	15.77	21.02	21.02
**55-59**	R	45.71	9.14	18.28	27.43	36.57	36.57	R	29.76	5.95	11.90	17.86	23.81	23.81
	L	42.16	8.43	16.86	25.30	33.73	33.73	L	27.81	5.56	11.12	16.69	22.25	22.25
**60-64**	R	40.59	8.12	16.24	24.35	32.47	32.47	R	26.35	5.27	10.54	15.81	21.08	21.08
	L	37.25	7.45	14.90	22.35	29.80	29.80	L	23.47	4.69	9.39	14.08	18.78	18.78
**65-69**	R	40.87	8.17	16.35	24.52	32.70	32.70	R	23.60	4.72	9.44	14.16	18.88	18.88
	L	36.57	7.31	14.63	21.94	29.26	29.26	L	23.38	4.68	9.35	14.03	18.70	18.70
**70-74**	R	37.48	7.50	14.99	22.49	29.98	29.98	R	25.84	5.17	10.34	15.50	20.67	20.67
	L	35.49	7.10	14.20	21.29	28.39	28.39	L	22.92	4.58	9.17	13.75	18.34	18.34
**75+**	R	32.76	6.55	13.10	19.66	26.21	26.21	R	19.40	3.88	7.76	11.64	15.52	15.52
	L	28.59	5.72	11.44	17.15	22.87	22.87	L	17.64	3.53	7.06	10.58	14.11	14.11

Gilbertson, L. and Barber-Lomax, S. (1994), ‘Power and Pinch Grip Strength Recorded Using the Hand-Held Jamar Dynamometer and B+L Hydraulic Pinch Guage: British Normative Data for Adults’, *British Journal of Occupational Therapy,* 57 (12), pp. 483-488.

© Copyright of Chelsea and Westminster NHS Foundation Trust (01/03/2010)

The Chelsea Critical Care Physical Assessment tool (CPAx)

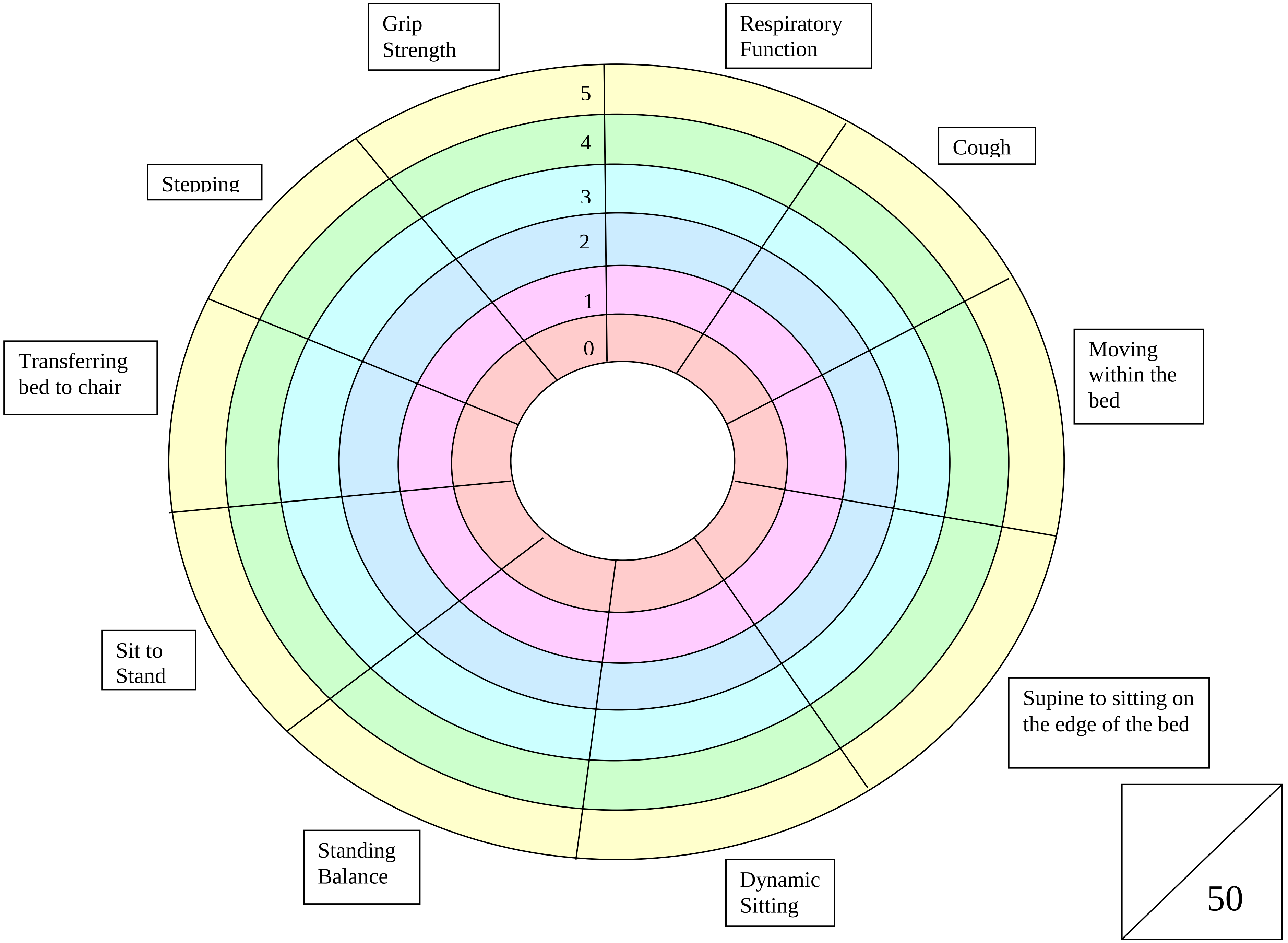

© Copyright of Chelsea and Westminster NHS Foundation Trust (01/03/2010)
